# The Dictator

**Published:** 1906-06

**Authors:** 


For Texas Medical Journal.

### The Dictator.

"Cromwell, I charge thee, fling away ambition."—*Cardinal Woolsey.*

Out in the wild and woolly West,
Where everything grows the very best—
At least, so all the good folks tell,
And I can't contradict them very well—
There lived a man *from o'er the seas,*
Who longed for fame and a life of ease.
He looked about, as men will do,
The same, perhaps as would I or you;
"This town, I know, is all very nice,
But, really, I think I prefer a slice
Of the cake prepared by that good Dame
Called by the poets the 'Goddess Fame.'
What can I do to win this prize
And quickly mount up to the skies?
A Homeopath is the thing for me,
And next an editor I will be!"

Now, this was well as far as it went—
For a doctor is almost an angel sent
From Heaven, on earth to do good deeds
And minister to another's needs.
His calling is noble, his work is grand,
There's no greater work in all the land!
He heals the sick and comforts the poor,
And his horse is found by the cottage door
As well as before the mansion of wealth,
Where he brings back the sick to a state of health.
He works all day and late at night,
With no thought of self or his weary plight.

But such honor was not enough, it seems
For this one man of greater dreams.
Said he, "I have but to take 'ethics' in hand,
And men will honor me all o'er the land.
I have only to gather them into the fold,
And drive them about like the sheep of old;
They are waiting around to be led by the nose!
I'll tackle the job—as a leader I'll pose.
Before the whole world they'll all cry aloud,
'Timmons the Great!' Our heads are all bowed
Before your greatness; be good to us, please!
Tell us, we implore, how we may choose
Such drugs and things as we may use.
For years we have lived and awaited your teaching!
How did we exist before your good preaching?
Our necks we bow low to take up your yoke—"
And I tell you it straight—in truth, 'twas no joke—
There were many good men who swallowed the bait,
And are still quite blind to their mournful fate.
But others there were who a "rodent did smell,"
And said to themselves, "We did very well
Before this great preacher of Homeo fame
Told us what we should do to cure the lame,
The blind and the deaf who sought our relief,
And sang our praises and had the belief;
We were not such fools as we seem to be
To the man who would lead us his greatness to see."
Some there were who, like D. Harum's steed,
Planted their feet and refused to take heed
Of the command of him who is known to fame
As the Octopus great, or some other name;
They cried, "Ha! ha!" and "It is to laugh!—
Does he think, indeed, we're the fatted calf?
We'll give him rope and he'll hang himself,
And be quietly laid away on the shelf."

This will be the end of the great good man
Who evolved from his brain the wonderful plan
Of making himself the leader of men,
And showing the world all over again
What a great big man could really do,
By blowing for himself a horn or two.
But one thing he forgot, 'tis plain to see—

The doctor who thought—like you and me—
The everyday practical busy one,
Whose work for many years was done
With glory, wide fame, and honor and praise,
And who never resorted to questionable ways—
Those of us who thought we really knew
A little of "ethics," and a thing or two
Before this great knowing man arose
To lead the practitioner by the nose.

But we'll lay this great man out of sight,
And forgotten he'll be, before the night.
Covered with dust and a mossy mould,
He'll lie like the Egyptian mummy of old;
Buried by those he thought to lead—
(Also quite incidentally to bleed)—
And we'll all go on in the self-same way,
As we've done for many and many a day;
And we'll go on and on till the setting sun—
In honor and peace, till our work is done.

DR. B——E.

No. — Twenty-seventh St., New York. February 6, 1906.

